# Correction: 2-Mercaptomethyl-thiazolidines use conserved aromatic–S interactions to achieve broad-range inhibition of metallo-β-lactamases

**DOI:** 10.1039/d3sc90150e

**Published:** 2023-08-15

**Authors:** Maria-Agustina Rossi, Veronica Martinez, Philip Hinchliffe, Maria F. Mojica, Valerie Castillo, Diego M. Moreno, Ryan Smith, Brad Spellberg, George L. Drusano, Claudia Banchio, Robert A. Bonomo, James Spencer, Alejandro J. Vila, Graciela Mahler

**Affiliations:** a Instituto de Biología Molecular y Celular de Rosario (IBR, CONICET-UNR) Ocampo and Esmeralda S2002LRK Rosario Argentina vila@ibr-conicet.gov.ar; b Laboratorio de Química Farmacéutica, Departamento de Química Orgánica, Facultad de Química, Universidad de la República (UdelaR) Avda. General Flores 2124 CC1157 Montevideo Uruguay gmahler@fq.edu.uy; c School of Cellular and Molecular Medicine, University of Bristol Biomedical Sciences Building, University Walk Bristol BS8 1TD UK; d Infectious Diseases Department, School of Medicine, Case Western Reserve University Cleveland OH USA; e Research Service, Louis Stokes Cleveland Department of Veterans Affairs Medical Center Cleveland OH USA; f Grupo de Resistencia Antimicrobiana y Epidemiología Hospitalaria, Universidad El Bosque Bogotá DC Colombia; g Instituto de Química de Rosario (IQUIR, CONICET-UNR) Suipacha 570 S2002LRK Rosario Argentina; h Los Angeles County and University of Southern California (LAC + USC) Medical Center Los Angeles CA USA; i Center for Pharmacometrics and Systems Pharmacology, Department of Pharmaceutics, College of Pharmacy, University of Florida Orlando FL USA; j Facultad de Ciencias Bioquímicas y Farmacéuticas, Universidad Nacional de Rosario S2002LRK Rosario Argentina; k Departments of Medicine, Pharmacology, Molecular Biology and Microbiology, Biochemistry, and Proteomics and Bioinformatics, Case Western Reserve University School of Medicine Cleveland OH USA; l Medical Service, GRECC, Louis Stokes Cleveland Department of Veterans Affairs Medical Center Cleveland OH USA; m CWRU-Cleveland VAMC Center for Antimicrobial Resistance and Epidemiology (Case VA CARES) Cleveland OH USA

## Abstract

Correction for ‘2-Mercaptomethyl-thiazolidines use conserved aromatic–S interactions to achieve broad-range inhibition of metallo-β-lactamases’ by Maria-Agustina Rossi *et al.*, *Chem. Sci.*, 2021, **12**, 2898–2908, https://doi.org/10.1039/D0SC05172A.

The authors regret that the CIF file of compound L-**1a** (CCDC: 2017238) was incorrect in the original article. The ESI of the original article has been updated to include the corrected CIF file of compound L-**1a**.

The Royal Society of Chemistry apologises for these errors and any consequent inconvenience to authors and readers.

## Supplementary Material

